# Multiple-gene panel analysis in a case series of 255 women with hereditary breast and ovarian cancer

**DOI:** 10.18632/oncotarget.16791

**Published:** 2017-04-03

**Authors:** Gianluca Tedaldi, Michela Tebaldi, Valentina Zampiga, Rita Danesi, Valentina Arcangeli, Mila Ravegnani, Ilaria Cangini, Francesca Pirini, Elisabetta Petracci, Andrea Rocca, Fabio Falcini, Dino Amadori, Daniele Calistri

**Affiliations:** ^1^ Biosciences Laboratory, Istituto Scientifico Romagnolo per lo Studio e la Cura dei Tumori (IRST) IRCCS, Meldola, Italy; ^2^ Romagna Cancer Registry, Istituto Scientifico Romagnolo per lo Studio e la Cura dei Tumori (IRST) IRCCS, Meldola, Italy; ^3^ Department of Medical Oncology, Ospedale Infermi, Rimini, Italy; ^4^ Unit of Biostatistics and Clinical Trials, Istituto Scientifico Romagnolo per lo Studio e la Cura dei Tumori (IRST) IRCCS, Meldola, Italy; ^5^ Department of Medical Oncology, Istituto Scientifico Romagnolo per lo Studio e la Cura dei Tumori (IRST) IRCCS, Meldola, Italy

**Keywords:** hereditary breast and ovarian cancer, multiple-gene panel, next-generation sequencing, bilateral breast cancer, cancer predisposition

## Abstract

As new genes predisposing to breast (BC) and ovarian cancer (OC) are constantly emerging, the use of panels of genes analyzed by Next-Generation Sequencing (NGS) is increasing in clinical diagnostics. The identification of a large number of new germline mutations allows for deeper knowledge of cancer predisposition, although raising many questions about patient management.

BC and OC patients recruited by our counseling service between 2012-2015 were included in this study. DNA was extracted from peripheral blood and a panel of 94 genes involved in hereditary tumors was analyzed by NGS. Patient clinical features of BC and OC and cancer family history were collected and compared to the patient genetic profile.

A total of 255 women were analyzed, 57 of whom had a pathogenic mutation in *BRCA1/2* genes, and 17 carried pathogenic mutations in other genes, such as *PALB2*, *ATM*, *BRIP1*, *RAD51D*, *MSH6*, *PPM1D*, *RECQL4*, *ERCC3*, *TSC2*, *SLX4* and other Fanconi anemia genes.

Patients with a pathogenic mutation in genes other than *BRCA1* and *BRCA2* showed no significant difference from the *BRCA1/2*-mutated carriers with respect to age at diagnosis and clinical features, suggesting that mutations in other genes could pose a high risk of cancer development.

These patients had a much higher percentage of bilateral breast cancer (BBC) and a lower rate of OC than *BRCA*-mutated patients and patients with no pathogenic mutations: as a consequence, the surveillance protocol should be customized to the patient genetic characteristics.

## INTRODUCTION

Breast cancer (BC) is the second most common cancer worldwide, and the most frequent cancer in women overall with about 1.7 million new cases diagnosed in 2012. BC is the second cause of cancer-related death in women in economically developed countries and the fifth worldwide [1].

Ovarian cancer (OC), is the fourth commonest cause of female cancer death in the developed world, also called “the silent killer” given the high mortality rate often due to late diagnosis [2].

About 10-30% of BCs and OCs shows a familial aggregation, but it is estimated that only 5-10% is hereditary, namely due to a genetic mutation which is transmitted to offspring [3, 4].

The main genes involved in hereditary breast and ovarian cancer (HBOC) are *BRCA1* [5], with 65% and 39% risk of developing BC and OC by the age of 70, respectively, and *BRCA2* [6], with 45% and 11% risk of developing BC and OC, respectively [7].

To date, many other genes have been associated to BC risk, such as *PALB2*, *TP53*, *ATM*, *BRIP1*, *CHEK2*, *CDH1*, *PTEN*, *STK11* [8, 9].

In the last few years the advent of Next-Generation Sequencing (NGS) has enabled the analysis of a greater number of genes with the advantage of lower costs and a wider access to molecular tests for patients with suspected genetic syndromes [10–13].

The discovery of new genes determining susceptibility to disease is crucial in oncology, as genetic transmission is more difficult to identify due to the frequent incomplete penetrance and the influence of the environment on genetics [14].

## RESULTS

We performed an NGS analysis of a panel of 94 genes involved in the main hereditary cancer syndromes (Supplementary Table 1) in a case series of 255 women.

The patient cohort included 227 (89.0%) patients with initial BC (median age 41 years) and 28 (11.0%) with initial OC (median age 49.5 years). BC and OC patient tumor characteristics are summarized in Tables 1 and 2, respectively.

**Table 1:** Clinical features and personal/family cancer history of BC patients

BREAST CANCER (BC)	All patients	Patients with *BRCA1/2* mutations	Patients with extra-*BRCA* mutations	Patients with no pathogenic mutations	*P*
	*N (%)*	*N (%)*	*N (%)*	*N (%)*	
**N. of patients**	227	48	17	162	
**Age at diagnosis, years**					
Median Age [Min-Max]	41 [25–79]	39 [25–70]	43 [26–74]	42 [25–79]	0.140
Missing	0	0	0	0	
**Histotype**					
In situ carcinoma	22 (10.14)	3 (6.52)	3 (17.65)	16 (10.39)	0.810
Invasive ductal carcinoma	148 (68.20)	33 (71.74)	10 (58.82)	105 (68.18)	
Invasive lobular carcinoma	26 (11.98)	6 (13.04)	3 (17.65)	17 (11.04)	
Other invasive histotypes	21 (9.68)	4 (8.70)	1 (5.88)	16 (10.39)	
Missing	10	2	0	8	
**Grading**					
Well-differentiated	18 (9.68)	0 (0.00)	1 (6.67)	17 (12.98)	0.005
Moderately differentiated	85 (45.70)	13 (32.50)	7 (46.67)	65 (49.62)	
Poorly differentiated	83 (44.62)	27 (67.50)	7 (46.67)	49 (37.40)	
Missing	41	8	2	31	
**Stage**					
0	22 (12.50)	3 (8.82)	3 (23.08)	16 (12.40)	0.375
I	92 (52.27)	15 (44.12)	5 (38.46)	72 (55.81)	
II	45 (25.57)	13 (38.24)	3 (23.08)	29 (22.48)	
III-IV	17 (9.66)	3 (8.82)	2 (15.38)	12 (9.30)	
Missing	51	14	4	33	
**Tumor invasiveness**					
In situ	22 (10.09)	3 (6.38)	3 (17.65)	16 (10.39)	0.420
Invasive	196 (89.91)	44 (93.62)	14 (82.35)	138 (89.61)	
Missing	9	1	0	8	
**Ki-67**					
High (≥14)	115 (70.55)	37 (90.24)	6 (75.00)	72 (63.16)	0.003
Low (<14)	48 (29.45)	4 (9.76)	2 (25.00)	42 (36.84)	
Missing	64	7	9	48	
**St Gallen subtype**					
Luminal A	29 (20.14)	1 (2.86)	2 (28.57)	26 (25.49)	0.005
Luminal B1	56 (38.89)	13 (37.14)	2 (28.57)	41 (40.20)	
Luminal B2	26 (18.06)	8 (22.86)	1 (14.29)	17 (16.67)	
Her2 positive	9 (6.25)	1 (2.86)	1 (14.29)	7 (6.86)	
Triple negative	24 (16.67)	12 (34.29)	1 (14.29)	11 (10.78)	
Missing	83	13	10	60	
**Second BC**					
No	175 (77.09)	36 (75.00)	9 (52.94)	130 (80.25)	0.036
Yes	52 (22.91)	12 (25.00)	8 (47.06)	32 (19.75)	
Median Age [Min-Max] ^a^	55 [32–82]	46 [37–70]	57 [36–77]	58.5 [32–82]	0.041
**Second OC**					
No	219 (96.48)	46 (95.83)	15 (88.24)	158 (97.53)	0.103
Yes	8 (3.52)	2 (4.17)	2 (11.76)	4 (2.47)	
Median Age [Min-Max]^a^	66.5 [51–77]	69 [68–70]	52.5 [51–54]	68.5 [55–77]	0.135
**Other tumors**					
No	205 (90.31)	45 (93.75)	17 (100.00)	143 (88.27)	0.254
Yes	22 (9.69)	3 (6.25)	0 (0.00)	19 (11.73)	
**BC/OC in I-degree relatives**					
No	81 (35.68)	17 (35.42)	10 (58.82)	54 (33.33)	0.113
Yes	146 (64.32)	31 (64.58)	7 (41.18)	108 (66.67)	
**BC/OC in I/II-degree relatives**					
No	43 (18.94)	10 (20.83)	7 (41.18)	26 (16.05)	0.039
Yes	184 (81.06)	38 (79.17)	10 (58.82)	136 (83.95)	
**Other cancers in I-degree relatives**					
No	144 (63.44)	33 (68.75)	11 (64.71)	100 (61.73)	0.670
Yes	83 (36.56)	15 (31.25)	6 (35.29)	62 (38.27)	
**Other cancers in I/II-degree relatives**					
No	81 (35.68)	22 (45.83)	7 (41.18)	52 (32.10)	0.193
Yes	146 (64.32)	26 (54.17)	10 (58.82)	110 (67.90)	

^a^ Median age, in years, refers to age at second cancer diagnosis

**Table 2:** Clinical features and personal/family cancer history of OC patients

OVARIAN CANCER (OC)	All patients	Patients with *BRCA1/2* mutations	Patients with extra-*BRCA* mutations	Patients with no pathogenic mutations	*P*
	*N (%)*	*N (%)*	*N (%)*	*N (%)*	
**N. of patients**	28	9	0	19	
**Age at diagnosis, years**					
Median Age [Min-Max]	49.5 [28–81]	50 [38–68]	-	47 [28–81]	0.640
Missing	0	0	-	0	
**Histotype**					
Serous carcinoma	18 (64.29)	7 (77.78)	-	11 (57.89)	0.700
Other malignant histotypes	7 (25.00)	2 (22.22)	-	5 (26.32)	
Borderline tumors	3 (10.71)	0 (0.00)	-	3 (15.79)	
Missing	0	0	-	0	
**Grading**					
Well-differentiated	2 (8.00)	0 (0.00)	-	2 (12.50)	0.772
Moderately differentiated	2 (8.00)	1 (11.11)	-	1 (6.25)	
Poorly differentiated	21 (84.00)	8 (88.89)	-	13 (81.25)	
Missing	3	0	-	3	
**Stage**					
0	0 (0.00)	0 (0.00)	-	0 (0.00)	0.343
I	7 (31.82)	1 (12.50)	-	6 (42.86)	
II	2 (9.09)	1 (12.50)	-	1 (7.14)	
III-IV	13 (59.09)	6 (75.00)	-	7 (50.00)	
Missing	6	1	-	5	
**Tumor invasiveness**					
Borderline	3 (10.71)	0 (0.00)	-	3 (15.79)	0.530
Invasive	25 (89.29)	9 (100.00)	-	16 (84.21)	
Missing	0	0	-	0	
**Second BC**					
No	21 (75.0)	7 (77.78)	-	14 (73.68)	1.000
Yes	7 (25.0)	2 (22.22)	-	5 (26.32)	
Median Age [Min-Max]^a^	55 [45–81]	58.5 [53–64]	-	55 [45–81]	1.000
**Other tumors**					
No	28 (100.00)	9 (100.00)	-	19 (100.00)	-
Yes	0 (0.00)	0 (0.00)	-	0 (0.00)	
**BC/OC in I-degree relatives**					
No	12 (42.86)	1 (11.11)	-	11 (57.89)	0.039
Yes	16 (57.14)	8 (88.89)	-	8 (42.11)	
**BC/OC in I/II-degree relatives**					
No	9 (32.14)	1 (11.11)	-	8 (42.11)	0.195
Yes	19 (67.86)	8 (88.89)	-	11 (57.89)	
**Other cancers in I-degree relatives**					
No	16 (57.14)	6 (66.67)	-	10 (52.63)	0.687
Yes	12 (42.86)	3 (33.33)	-	9 (47.37)	
**Other cancers in I/II-degree relatives**					
No	12 (42.86)	5 (55.56)	-	7 (36.84)	0.432
Yes	16 (57.14)	4 (44.44)	-	12 (63.16)	

^a^ Median age, in years, refers to age at second cancer diagnosis

Of the 227 BC patients, 52 (22.9%) had bilateral breast cancer (BBC), 8 (3.5%) had subsequent OC and 22 (9.7%) had other malignancies (reported as “Second BC”, “Second OC” and “Other tumors”, respectively, in Table 1).

Of the 28 OC patients, 7 (25.0%) had subsequent BC (reported as “Second BC” in Table 2). None (0.0%) presented other malignancies (reported as “Other tumors” in Table 2).

The molecular analysis of the 255 patients showed a mean target coverage of 399,7X and a 95.5% mean percentage of target covered >50X.

We focused at first on the *BRCA* mutation status of patients.

According to the databases and guidelines (see Materials and Methods), 57 (22.4%) patients had a pathogenic/likely-pathogenic mutation in *BRCA* genes, in particular 31 (12.2%) had a *BRCA1* mutation, 25 (9.8%) had a *BRCA2* mutation and 1 (0.4%) had pathogenic mutations in both *BRCA1* and *BRCA2* (Supplementary Table 2).

We then observed the mutations in the remaining 92 genes of the panel.

The analysis revealed 23 pathogenic/likely-pathogenic mutations in 14 genes in 21/255 (8.2%) patients (Supplementary Table 3). Out of these 21 patients, 4 were also *BRCA*-positive and 17 *BRCA*-negative.

We finally analyzed the 181 (71.0%) patients with pathogenic mutations in neither *BRCA1/2* nor other genes, showing 23,882 exonic and splicing (± 5bp) variants.

The frequencies present in 1000Genomes, Esp6500 and Exac03 databases were used to exclude polymorphic variants.

Among the remaining 1,026 variants with frequency <1% or n/a, we worked on the missense variants with PolyPhen-2 HVar and SIFT to assess their possible role in cancer development.

### *BRCA* mutations and patient characteristics

We identified 32 pathogenic/likely-pathogenic mutations in *BRCA1* gene and 26 in *BRCA2* gene (Supplementary Table 2).

Most of the 31 patients with a *BRCA1* pathogenic/likely-pathogenic mutation had BC: 23 (74.2%) had initial BC, 6 (26.1%) of whom BBC, and 1 (4.3%) subsequent OC. The remaining 8 (25.8%) had initial OC, 2 (25.0%) of whom had subsequent BC.

Also most of the 25 patients with a *BRCA2* pathogenic/likely-pathogenic mutation had BC: 24 (96.0%) had initial BC, 6 (25.0%) of whom BBC, and 1 (4.2%) had subsequent OC. Only 1 (4.0%) patient had initial OC.

The only patient with pathogenic mutations in both *BRCA1* and *BRCA2* had BC.

The clinical features of the 31 *BRCA1*-mutated patients were compared with those of the 25 *BRCA2*-mutated patients with no statistically significant differences, except for the grading of BC with a higher number of poorly differentiated tumors in *BRCA1*-mutated patients (Supplementary Tables 4 and 5). The two groups were thus treated as one group including the single patient with both *BRCA1* and *BRCA2* genes mutated (Tables 1 and 2).

Median age at the onset of BC was 39 years for initial BC and 46 for subsequent BC. Median age at the onset of OC was 50 years.

The number of triple-negative BCs was significantly higher in *BRCA*-positive patients (34.3%) than in *BRCA*-negative patients (11.0%).

The BC/OC family history in I- and II-degree relatives was significantly higher in *BRCA*-mutated patients and *BRCA*-wild type patients with BC than in patients with mutations in extra-*BRCA* genes (*P*=0.039, Table 1).

Also *BRCA*-mutated patients with OC had a higher BC/OC family history in I-degree relatives than *BRCA*-wild type patients (*P*=0.039, Table 2).

### Extra-*BRCA* mutations and patient characteristics

Among the 23 pathogenic/likely-pathogenic mutations identified (Supplementary Table 3), 1 deletion in *ERCC3* gene was found in 1 patient with a pathogenic mutation in *BRCA1*, and 3 mutations (1 deletion in *FANCA* gene, 1 deletion in *BRIP1* gene and 1 nonsense mutation in *ATM*) were found in 3 patients with a pathogenic mutation in *BRCA2*.

The remaining 19 pathogenic/likely-pathogenic mutations found in 17 *BRCA1/2* wild-type patients included 6 mutations in *PALB2* (3 deletions and 3 nonsense mutations), 2 in *ATM* (1 deletion and 1 insertion), 2 in *FANCL* (1 insertion and 1 nonsense mutation), 1 deletion in *BRIP1*, 1 nonsense mutation in *FANCM*, 1 deletion in *FANCI*, 1 deletion in *SLX4*, 1 nonsense mutation in *MSH6*, 1 nonsense mutation in *RAD51D*, 1 deletion in *PPM1D*, 1 deletion in *RECQL4*, and 1 deletion in *TSC2*.

The *FANCL* insertion and 1 of the *PALB2* nonsense mutations were both present in 1 patient; the *BRIP1* deletion and the *SLX4* deletion were both present in another patient.

All 23 variants had either <1% or n/a frequency in the population (1000Genomes, Esp6500 and Exac03 databases) and where checked in dbSNP and ClinVar databases (Supplementary Table 3 and Materials and Methods).

All 17 (100.0%) patients had initial BC, 8 (47.1%) of whom had BBC and 2 (11.8%) had subsequent OC. Median age at the onset of BC was 43 years for initial BC, 57 for subsequent BC and 52.5 for subsequent OC (Table 1). None of these patients had cancers other than BC or OC.

**Figure 1:**
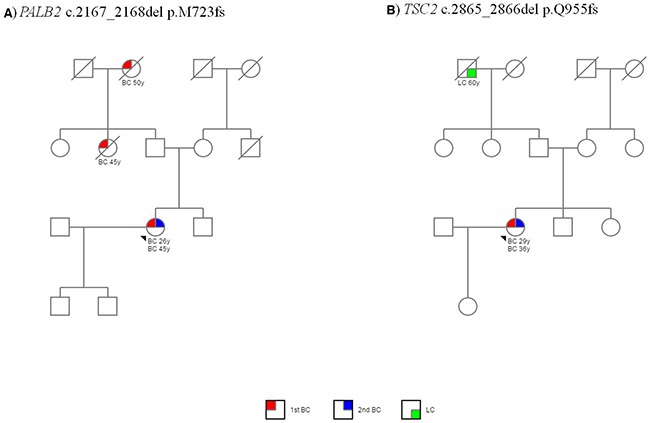
Pedigrees of two patients with a mutation in extra-*BRCA* genes (**A**) Pedigree of patient A243 with c.2167_2168del p.M723fs mutation in *PALB*2 gene. (**B**) Pedigree of patient A790 with c.2865_2866del p.Q955fs mutation in *TSC2* gene. The probands are indicated by arrowheads. Cancer type and age at cancer diagnosis are indicated in the legend: 1st BC, first breast cancer; 2nd BC, second breast cancer; LC, lung cancer. Symbols: squares, males; circles, females; quadrant shading, cancer affected; slash through square or circle, deceased.

The family history of these patients included BC/OC and other types of cancer, as shown in the pedigrees of 2 patients with *PALB2* and *TSC2* mutations (Figure 1A and 1B).

### Characteristics of patients with no pathogenic mutations

Out of 181 patients with no pathogenic mutations, 162 (89.5%) had initial BC, of whom 32 (19.8%) had BBC and 4 (2.5%) had subsequent OC. Median age was 42 years at onset of initial BC, 58.5 for subsequent BC, and 68.5 for subsequent OC (Table 1).

The remaining 19 (10.5%) had initial OC, 5 (26.3%) of whom had subsequent BC. Median age was 47 years at onset of initial OC, and 55 for subsequent BC (Table 2).

Among these 181 patients we identified 1,026 variants with <1% or n/a population frequency: 379 (36.9%) were synonymous mutations, 631 (61.5%) missense mutations, 6 (0.6%) nonframeshift deletions, 2 (0.2%) nonframeshift insertions, and 8 (0.8%) splicing mutations, with a total of 674 unique variants in 92 genes, and an average of 6 variants per patient.

Among the 1,026 rare variants identified, we worked on the 631 missense variants using functional effect prediction tools PolyPhen-2 HVar and SIFT, dividing the 181 patients into 3 categories: the first group (70 patients) with at least 1 mutation classified as damaging by both PolyPhen-2 Hvar and SIFT, the second group (26 patients) with mutations discordantly classified, and the third group (85 patients) with mutations classified as benign. No statistically significant differences were found between the three groups (Supplementary Tables 6 and 7).

## DISCUSSION

Current clinical genetic tests for BC and OC risks have been based on the analysis of *BRCA1* and *BRCA2* genes only, despite new evidence of a higher number of genes eligible for testing [15].

Given the considerable amount of genes whose mutations have a role in determining a broad spectrum of tumors, we used a gene panel including almost all the genes involved in the main hereditary cancer syndromes.

To our knowledge, this is the first large Italian study on the sequencing of a multiple-gene panel for cancer predisposition and one of the widest genetic studies on HBOC for both the number of genes analyzed and the number of recruited patients [10, 12, 13, 16, 17].

We detected a total of 81 pathogenic/likely-pathogenic mutations in 74/255 (29.0%) patients, 32 (39.5%) in *BRCA1*, 26 (32.1%) in *BRCA2* and 23 (28.4%) in other genes. The 23 mutations in the other genes were present in 21 patients, 17 of whom were negative for *BRCA* genes; some of these genes were not clearly correlated to BC.

The 57 patients with *BRCA1/2* pathogenic mutations have already been included in a surveillance protocol according to the F.O.N.Ca.M. (Forza Operativa Nazionale sul Carcinoma Mammario) guidelines [18] and the genetic test has been performed on their consenting relatives.

The BC characteristics of *BRCA*-mutated patients corresponded to what is described in literature [19, 20], with a significant higher number of poorly differentiated tumors (*P*=0.005), a significant number of triple-negative cancers (*P*=0.005) and higher Ki-67 expression (*P*=0.003) than in other patients (Table 1), which are all signs of the greater aggressiveness of the malignancy.

*BRCA*-mutated patients, compared to other patients, developed BC at a younger age, especially second BC (*P*=0.041), and had a higher family history of BC/OC, especially for I-degree relatives of OC patients (*P*=0.039), which are both predictable results given the higher penetrance of mutations in *BRCA1/2* genes (Tables 1 and 2).

Thirteen patients had alterations in *ATM*, *BRIP1*, *PALB2*, *PPM1D* and *RAD51D* genes, which are known to be associated with an increased risk of BC, even if they are considered moderate penetrant genes [8]. Guidelines for the clinical management of mutation carriers are still unavailable.

Thanks to the discovery of these mutations, these patients and their families are eligible for further studies on the development of malignancies in mutation carriers over time, which combine our case series with those of other institutes with the same type of patients.

*PALB2*, the most frequent mutated gene after *BRCA1* and *BRCA2* in our case series, is worth mentioning. As recently reported by Antoniou and colleagues [21], *PALB2* gene has been proven the most important BC predisposition gene after *BRCA1* and *BRCA2*.

We found 6 patients negative for *BRCA1/2* mutations with a pathogenic mutation in *PALB2* gene, 4 (66.7%) of whom had BBC. These data further highlighted both the high risk of BC associated with these mutations and the importance of introducing the *PALB2* gene in standard genetic analysis protocols for patients with suspected hereditary BC syndrome.

Two patients (A482 and A806) were carriers of frameshift mutations in *BRIP1* gene, whose truncating mutations have been recently excluded from having a role in BC risk [22]. This had no effect on their assigned category, as each patient had another deleterious mutation (*BRCA2* and *SLX4* respectively).

We also found 1 patient with a pathogenic mutation in *MSH6* gene, associated to Lynch syndrome, a colorectal cancer syndrome whose correlation with BC is still debated [23]: this finding will allow for appropriate genetic counseling and the extension of the genetic test to the relatives. The surveillance protocol for these patients must take the cancer family history and the cancer risk given by the mutation into account. In the case of *MSH6* mutation, the family will undergo a surveillance protocol including screening for BC, as it is the only cancer type present in the family, and screening for colon cancer, according to the Lynch syndrome guidelines [24], as the risk for colon cancer in *MSH6* mutation carriers cannot be ignored.

Six patients had mutations in *FANCA*, *FANCI*, *FANCL*, *FANCM* and *SLX4* genes, which are involved in Fanconi anemia (FA). FA is a recessive genetic disorder characterized by multiple congenital abnormalities, bone marrow failure and susceptibility to cancer, occurring when both the alleles of one of the FA genes are mutated. Monoallelic mutations of some FA genes have been associated to BC risk [25, 26], and biallelic mutations in *BRCA2* have been associated to FA [27]. These observations suggest that biallelic mutations of these genes may result in FA and that monoallelic mutations can pose a risk of BC. Further studies are necessary to confirm such association and to assess the actual risk for the patients.

Finally, we found 3 pathogenic/likely-pathogenic mutations in *ERCC3*, *RECQL4* and *TSC2* genes, encoding transcription factors and tumor suppressors.

Although mutations in these genes are not clearly associated with BC, a role in the predisposition to BC cannot be excluded since they are involved in the major cancer pathways.

Specific mutations in *ERCC3* and *RECQL*, a homologue of *RECQL4*, have also been identified in families with multiple BC cases [28, 29].

The management of these patients still remains problematic. Only further studies on larger case series will determine the factual cancer risk for the mutation carriers.

It is important to underline that the pathogenicity of the identified variants based on the guidelines [30] refers to their potential role in cancer development, not to their causality of BC, as there might be other variants in genes not analyzed in the present study.

We detected a much higher percentage (47.1%) of BBCs in patients with pathogenic mutations in non-*BRCA* genes than in *BRCA1*- (26.1%) and *BRCA2*-positive patients (25.0%) (*P*=0.036), despite their older age at onset (Table 1). This suggests a high penetrance and a high risk of BC for the carriers; the pathogenic mutations in genes other than *BRCA1/2* do not appear to be linked to OC, since all these patients have BC, only 2 of whom developed OC as second tumor.

These results underscore the importance of a multigenic approach for identifying the genetic cause in a greater number of cases than with a targeted analysis on *BRCA1/2* genes. It also allows accurate patient monitoring for developing surveillance programs customized to their genetic characteristics.

Another remarkable feature is the lower family history of BC/OC in I- and II-degree relatives (*P*=0.039) than for both the *BRCA*-mutated patients and the patients with no pathogenic mutations (Table 1). Although this result should be verified in larger studies, we hypothesize that it might be due to the fact that these patients have a heterogeneous cancer family history, which includes other types of cancer.

No clear pathogenic mutation was identified in 181/255 (71.0%) patients. We thus studied the 1,026 rare variants identified in order to assess whether they could contribute to cancer risk.

NGS-based studies lead to the identification of many non-easily classifiable variants. Several techniques can now be used to determine pathogenicity of mutations [31], yet quick, efficient and accurate methods for classifying variants are needed for translating the information to clinical practice.

The bioinformatic tools for the prediction of pathogenicity used in this study seemed irrelevant for discriminating higher risk from lower risk patients. This may be due to the fact that the bioinformatic prediction method used in the present work is based only on two different tools, which can be insufficient to highlight clinicopathological differences among the patients. Moreover, the multifactorial nature of the disease and the possible presence of alterations in genes other than those analyzed in this study could explain this result. Some of the identified variants, however, may increase BC and OC risk, whose determination is difficult due to the limited number of carriers and the interference of other genetic and environmental factors.

The interpretation of the potential role in disease development of the great number of variants identified by NGS-based studies remains one of the major future challenges.

## MATERIALS AND METHODS

### Ethics statement

Investigation was conducted in accordance with ethical standards, the Declaration of Helsinki and national and international guidelines. It was also approved by the authors’ institutional review board.

### Patients and samples

Patients referring to genetic counseling at the Cancer Prevention Unit of the Morgagni-Pierantoni Hospital (Forlì-Italy) in the years 2012-2015 with a history of BC and/or OC were included in the study.

The 255 patients were selected according to the F.O.N.Ca.M guidelines [18], based on the age at BC/OC onset and on the number of cancer cases in I- and II-degree relatives.

The study was performed in accordance with the Good Clinical Practice and the Declaration of Helsinki, and approved by the IRST Ethics Committee (CE IRST IRCCS-AVR, protocol 2207/2012).

Information about age at diagnosis, histotype, grading, stage, tumor invasiveness and receptor status was collected. BC subtype classification, based on receptor status, was established according to the St Gallen guidelines [32].

Information about a second BC and/or OC or other malignancies and the cancer family history in I- and II-degree relatives was also collected.

After obtaining informed consent from patients, we collected peripheral blood samples.

Genomic DNA was extracted from blood using the QIAamp DNA mini kit (Qiagen) and quantified using the Qubit dsDNA BR Assay Kit (Thermo Fisher Scientific).

### Sequencing

Sequencing libraries were created using 50 ng of genomic DNA and the enrichment protocol Trusight Cancer (Illumina) for simultaneous sequencing of a panel of 94 genes (Supplementary Table 1).

The panel covers a total of 355 kb and includes the entire coding regions of the 94 genes and the flanking introns (50bp upstream and downstream each exon).

The sequencing was performed using the MiSeq platform (Illumina) with MiSeq Reagent Kit v2 configured 2×150 cycles, according to the manufacturer's instructions.

The Trusight Cancer kit had been previously validated in our laboratory on a case series of 50 cases with known *BRCA1/2* mutations identified by Sanger sequencing.

### Data analysis and variant calling

Raw de-multiplexed reads from the MiSeq sequencer were aligned to the reference human genome (UCSC-Build37/hg19) using the Burrows–Wheeler algorithm [33], running in paired-end mode. To ensure good call quality and to reduce the number of false positives, samples underwent Base Quality Score Recalibration (BQSR), using the Genome Analysis Toolkit GATK, version 3.2.2 [34]. After BQSR, sequences around regions with insertions and deletions (indels) were realigned locally with GATK. MarkDuplicates [35] was used to remove duplicate read-pairs arisen as artifacts during either polymerase chain reaction amplification or sequencing. For variant analysis Unified Genotyper of GATK was used to search for SNVs and indels. Genomic and functional annotations of detected variants were made by Annovar [36]. Coverage statistics was performed by DepthOfCoverage utility of GATK. BASH and R custom scripts were used to obtain the list of low coverage (<50X) regions per sample. The regions under this threshold were considered not evaluable. The potential impact of amino acid changes (MAPP P value) was assessed with PolyPhen-2 HVAR [37] and SIFT [38].

### *BRCA1/2* analysis

*BRCA1/2* regions covered <50X were amplified by standard polymerase chain reaction (PCR). PCR products were sequenced using the BigDye terminator v.3.1 cycle sequencing kit (Thermo Fisher Scientific) on an ABI-3130 Genetic analyzer (Applied Biosystems).

To complete the analysis on the *BRCA1/2* genes and identify gross deletions/insertions not detectable by sequencing, we performed the Multiplex Ligation-dependent Probe Amplification (MLPA) with BRCA1-P002 and BRCA2-P045 kits (MRC Holland). MLPA results were analyzed with Coffalyser software (MRC Holland).

### Confirmation of mutations

All the mutations of classes 3-5 identified in *BRCA1* and *BRCA2* genes were confirmed by Sanger sequencing with the same protocol used for the uncovered regions.

All the deleterious mutations (classes 4-5) identified in the other genes were confirmed by a second NGS analysis.

### Variant classification

Genetic variants identified in this work were divided into 5 classes according to the IARC recommendations [39].

The classification of *BRCA1/2* variants was performed using the main *BRCA* mutation databases: Breast Cancer Information Core (BIC) [40], *BRCA* Share (formerly Universal Mutation Database) [41] and Leiden Open Variation Database (LOVD) [42].

Sequence variants in the remaining 92 genes were classified using dbSNP [43] and ClinVar [44] databases.

Variants not present in any of these databases were classified on the basis of their characteristics. Only mutations introducing a premature stop codon (frameshift and nonsense) and gross deletions were considered pathogenic/likely-pathogenic and classified in accordance with the guidelines of the American College of Medical Genetics (ACMG) [30].

### Statistical analysis

Patient characteristics and sequencing results were tabulated, with descriptive statistics including median and range for continuous data, and natural frequencies and percentages for categorical data. Proportions were compared using either the Pearson Chi-square test or the Fisher Exact test, as appropriate. The Wilcoxon-Mann Whitney or the Kruskal-Wallis test, as appropriate, were used for the continuous variables.

All P values were two-tailed. Analyses were performed using STATA Release 14.0.

## SUPPLEMENTARY MATERIALS TABLES















## Abbreviations

BC: breast cancer
OC: ovarian cancer
NGS: Next-Generation Sequencing
BBC: bilateral breast cancer
HBOC: hereditary breast and ovarian cancer
F.O.N.Ca.M.: Forza Operativa Nazionale sul Carcinoma Mammario
FA: Fanconi anemia
BQSR: Base Quality Score Recalibration
PCR: polymerase chain reaction
MLPA: Multiplex Ligation-dependent Probe Amplification
BIC: Breast Cancer Information Core
LOVD: Leiden Open Variation Database
ACMG: American College of Medical Genetics.
