# Supplementary material for: Multiple-gene panel analysis in a case series of 255 women with hereditary breast and ovarian cancer
Source: Oncotarget. 2017 Apr 3;8(29):47064–75. doi: 10.18632/oncotarget.16791 (PMC5564544; doi:10.18632/oncotarget.16791)
Supplement: Supplementary file 1 [file oncotarget-08-47064-s001.pdf]

# Multiple-gene panel analysis in a case series of 255 women with hereditary breast and ovarian cancer

## SUPPLEMENTARY TABLES

**Supplementary Table 1: List of the 94 genes in the Trusight Cancer panel (Illumina)**

| | | | | | | | | | |
|---|---|---|---|---|---|---|---|---|---|
| *AIP* | *ALK* | *APC* | *ATM* | *BAP1* | *BLM* | *BMPR1A* | *BRCA1* | *BRCA2* | *BRIP1* |
| *BUB1B* | *CDC73* | *CDH1* | *CDK4* | *CDKN1C* | *CDKN2A* | *CEBPA* | *CEP57* | *CHEK2* | *CYLD* |
| *DDB2* | *DICER1* | *DIS3L2* | *EGFR* | *EPCAM* | *ERCC2* | *ERCC3* | *ERCC4* | *ERCC5* | *EXT1* |
| *EXT2* | *EZH2* | *FANCA* | *FANCB* | *FANCC* | *FANCD2* | *FANCE* | *FANCF* | *FANCG* | *FANCI* |
| *FANCL* | *FANCM* | *FH* | *FLCN* | *GATA2* | *GPC3* | *HNF1A* | *HRAS* | *KIT* | *MAX* |
| *MEN1* | *MET* | *MLH1* | *MSH2* | *MSH6* | *MUTYH* | *NBN* | *NF1* | *NF2* | *NSD1* |
| *PALB2* | *PHOX2B* | *PMS1* | *PMS2* | *PRF1* | *PRKAR1A* | *PTCH1* | *PTEN* | *RAD51C* | *RAD51D* |
| *RB1* | *RECQL4* | *RET* | *RHBDF2* | *RUNX1* | *SBDS* | *SDHAF2* | *SDHB* | *SDHC* | *SDHD* |
| *SLX4* | *SMAD4* | *SMARCB1* | *STK11* | *SUFU* | *TMEM127* | *TP53* | *TSC1* | *TSC2* | *VHL* |
| *WRN* | *WT1* | *XPA* | *XPC* | | | | | | |

**Supplementary Table 2: List of *BRCA1* and *BRCA2* pathogenic/likely-pathogenic mutations detected in 57 patients.**

See Supplementary File 1

**Supplementary Table 3: List of the pathogenic/likely-pathogenic mutations in extra-*BRCA* genes detected in 21 patients.**

See Supplementary File 2

**Supplementary Table 4: Clinical features and personal/family cancer history of BC patients with *BRCA1* and *BRCA2* pathogenic mutations.**

See Supplementary File 3

**Supplementary Table 5: Clinical features and personal/family cancer history of OC patients with *BRCA1* and *BRCA2* pathogenic mutations.**

See Supplementary File 4

**Supplementary Table 6: Clinical features and personal/family cancer history of BC patients with no pathogenic mutations, classified on the basis of PolyPhen HVAR and SIFT predictions.**

See Supplementary File 5

**Supplementary Table 7: Clinical features and personal/family cancer history of OC patients with no pathogenic mutations, classified on the basis of PolyPhen HVAR and SIFT predictions.**

See Supplementary File 6
