# Supplementary material for: Multiple-gene panel analysis in a case series of 255 women with hereditary breast and ovarian cancer
Source: Oncotarget. 2017 Apr 3;8(29):47064–75. doi: 10.18632/oncotarget.16791 (PMC5564544; doi:10.18632/oncotarget.16791)
Supplement: Supplementary file 2 [file oncotarget-08-47064-s002.docx]

Supplementary Table S2: List of *BRCA1* and *BRCA2* pathogenic/likely-pathogenic mutations detected in 57 patients.

| **Sample ID** | **Cancer (age)^a^** | **Gene** | **chr** | **start** | **end** | **ref** | **alt** | **Mutation type** | **HGVS^b^** | **depth** | **VAF^c^** | **IARC class** | **BRCA Share-BIC-LOVD^d^** | **dbSNP/ClinVar^e^** |
| --- | --- | --- | --- | --- | --- | --- | --- | --- | --- | --- | --- | --- | --- | --- |
| A004 | IDC (33y), IDC (37y) | *BRCA2* | 13 | 32954142 | 32954142 | A | G | splicing | NM_000059: exon 24, c.9118-2A>G | 321 | 0.49 | class 5 | Pathogenic | rs81002862/Pathogenic |
| A034 | IDC (43y) | *BRCA2* | 13 | 32953453 | 32953453 | G | A | splicing | NM_000059: exon 22, c.8755-1G>A | 368 | 0.42 | class 4 | VUS/Pathogenic | rs81002812/Likely pathogenic |
| A154 | IDC (32y) | *BRCA1* | 17 | 41197784 | 41197784 | G | A | nonsense | NM_007294: exon 24, c.5503C>T p.Arg1835Ter | 336 | 0.38 | class 5 | Pathogenic | rs41293465/Pathogenic |
| A194 | IDC (51y), IDC (54y) | *BRCA1* | 17 | 41245647 | 41245647 | - | GTGGGCTTAGATTT | frameshift insertion | NM_007294: exon 11, c.1887_1900dupAAATCTAAGCCCAC p.Pro634fs | 583 | 0.18 | class 5 | - | rs886039977/Pathogenic |
| A200 | OPC (66y) | *BRCA2* | 13 | 32930609 | 32930609 | C | T | nonsense | NM_000059: exon 15, c.7480C>T p.Arg2494Ter | 545 | 0.43 | class 5 | Pathogenic | rs80358972/Pathogenic |
| A207 | IDC (48y) | *BRCA1* | 17 | 41228505 | 41228505 | C | A | missense | NM_007294: exon 14, c.4484G>T p.Arg1495Met | 1125 | 0.52 | class 5 | Pathogenic | rs80357389/Pathogenic |
| A236 | BC (37y) | *BRCA2* | 13 | 32911684 | 32911687 | AATT | - | frameshift deletion | NM_000059: exon 11, c.3192_3195delAATT p.Ser1064fs | 436 | 0.37 | class 5 | Pathogenic | rs80359375/Pathogenic |
| A284 | IDC (39y) | *BRCA1* | 17 | 41209079 | 41209079 | - | G | frameshift insertion | NM_007294: exon 20, c.5266dupC p.Gln1756fs | 1340 | 0.33 | class 5 | Pathogenic | rs80357906/Pathogenic |
| A305 | ILC (44y) | *BRCA2* | 13 | 32890665 | 32890665 | G | A | splicing | NM_000059: exon 2, c.67+1G>A | 740 | 0.45 | class 5 | Pathogenic | rs81002796/Pathogenic |
| A311 | IDC (27y) | *BRCA1* | 13 | - | - | exons 1-2 | - | gross deletion | NM_007294: exons 1-2, c.1-?_80+?del p.? | - | - | class 5 | Pathogenic | - |
| A391 | IDC (41y) | *BRCA1* | 17 | 41226450 | 41226450 | G | A | nonsense | NM_007294: exon 15, c.4573C>T p.Gln1525Ter | 725 | 0.52 | class 5 | - | rs886040237/Pathogenic |
| A392 | IDC (34y) | *BRCA1* | 17 | - | - | exon 24 | - | gross deletion | NM_007294: exon 24, c.5468-?_5592+?del p.? | - | - | class 5 | Pathogenic | - |
| A396 | IDC (39y) | *BRCA2* | 13 | 32912354 | 32912357 | ATAA | - | frameshift deletion | NM_000059: exon 11, c.3860_3863delATAA p.Asn1287fs | 488 | 0.49 | class 5 | Pathogenic | rs80359410/Pathogenic |
| A407 | ILC (39y) | *BRCA1* | 17 | 41246566 | 41246567 | AT | - | frameshift deletion | NM_007294: exon 11, c.981_982del p.Thr327fs | 925 | 0.47 | class 5 | Pathogenic | rs80357772/Pathogenic |
| A414 | IDC (30y) | *BRCA2* | 13 | 32921023 | 32921023 | - | T | frameshift insertion | NM_000059: exon 13,c.6998dupT p.Pro2334fs | 272 | 0.30 | class 5 | Pathogenic | rs754611265/Pathogenic |
| A420 | OSC (41y) | *BRCA1* | 17 | 41209079 | 41209079 | - | G | frameshift insertion | NM_007294: exon 20, c.5266dupC p.Gln1756fs | 1218 | 0.18 | class 5 | Pathogenic | rs80357906/Pathogenic |
| A427 | IDC (26y) | *BRCA1* | 17 | 41215920 | 41215920 | G | T | missense | NM_007294: exon 18, c.5123C>A p.Ala1708Glu | 319 | 0.42 | class 5 | Pathogenic | rs28897696/Pathogenic |
| A433 | IDC (34y) | *BRCA2* | 13 | 32914894 | 32914898 | TAACT | - | frameshift deletion | NM_000059: exon 11, c.6402_6406delTAACT p.Asn2134fs | 633 | 0.46 | class 5 | Pathogenic | rs80359584/Pathogenic |
| A467 | IDC (45y) | *BRCA1* | 17 | 41245251 | 41245252 | CT | - | frameshift deletion | NM_007294: exon 11, c.2296_2297del p.Ser766fs | 433 | 0.42 | class 5 | Pathogenic | rs80357780/Pathogenic |
| A469 | IDC (37y) | *BRCA1* | 17 | 41245861 | 41245861 | G | A | nonsense | NM_007294: exon 11, c.1687C>T p.Gln563Ter | 434 | 0.49 | class 5 | Pathogenic | rs80356898/Pathogenic |
| A482 | IDC (37y), IDC (37y) | *BRCA2* | 13 | 32903605 | 32903606 | TG | - | frameshift deletion | NM_000059: exon 8, c.658_659delGT p.Val220fs | 554 | 0.41 | class 5 | Pathogenic | rs80359604/Pathogenic |
| A497 | OSC (50y) | *BRCA1* | 17 | 41246764 | 41246764 | G | - | frameshift deletion | NM_007294: exon 11, c.784delC p.Gln262fs | 362 | 0.52 | class 4 | VUS | rs886040318/Pathogenic |
| A500 | OAC (38y) | *BRCA1* | 17 | 41251790 | 41251790 | A | T | splicing | NM_007294: exon 8, c.547+2T>A | 343 | 0.43 | class 5 | Pathogenic | rs80358047/Pathogenic |
| A503 | IDC (25y) | *BRCA2* | 13 | 32911143 | 32911146 | CAGA | - | frameshift deletion | NM_000059: exon 11, c.2653_2656delGACA p.Asp885fs | 484 | 0.48 | class 5 | Pathogenic | rs80359340/Pathogenic |
| A525 | IDC (48y) | *BRCA2* | 13 | 32914529 | 32914529 | A | - | frameshift deletion | NM_000059: exon 11, c.6039delA p.Val2014fs | 473 | 0.43 | class 5 | - | rs876660637/Pathogenic |
| A575 | OSC (40y), IDC (64y) | *BRCA1* | 17 | - | - | exon 20 | - | gross deletion | NM_007294: exon 20, c.5194-?_5277+?del p.? | - | - | class 5 | Pathogenic | Pathogenic |
| A576 | IDC (37y), IDC (54y) | *BRCA1* | 17 | 41228505 | 41228505 | C | A | missense | NM_007294: exon 14, c.4484G>T p.Arg1495Met | 714 | 0.54 | class 5 | Pathogenic | rs80357389/Pathogenic |
| A598 | ILC (28y) | *BRCA1* | 17 | 41219660 | 41219664 | ATTAG | - | frameshift deletion | NM_007294: exon 17, c.5035_5039del p.Leu1679fs | 481 | 0.43 | class 5 | Pathogenic | rs80357623/Pathogenic |
| A601 | OSC (68y) | *BRCA1* | 17 | 41243789 | 41243792 | AGAC | - | frameshift deletion | NM_007294: exon 11, c.3756_3759del p.Leu1252fs | 314 | 0.34 | class 5 | Pathogenic | rs80357868/Pathogenic |
| A606 | ILC (34y) | *BRCA2* | 13 | 32915083 | 32915084 | TG | - | frameshift deletion | NM_000059: exon 11, c.6591_6592delTG p.Thr2197fs | 226 | 0.42 | class 5 | Pathogenic | rs80359605/Pathogenic |
| A616 | IDC (41y), IDC (41y) | *BRCA1* | 17 | 41243789 | 41243792 | AGAC | - | frameshift deletion | NM_007294: exon 11, c.3756_3759del p.Leu1252fs | 305 | 0.48 | class 5 | Pathogenic | rs80357868/Pathogenic |
| A619 | IDC (32y) | *BRCA2* | 13 | 32912338 | 32912339 | TG | - | frameshift deletion | NM_000059: exon 11, c.3847_3848delGT p.Val1283fs | 470 | 0.48 | class 5 | Pathogenic | rs746229647/Pathogenic |
| A620v | DCIS (41y) | *BRCA2* | 13 | 32907421 | 32907421 | A | - | frameshift deletion | NM_000059: exon 10, c.1806delA p.Gly602fs | 465 | 0.50 | class 5 | Pathogenic | rs80359307/Pathogenic |
| A623 | IDC (53y) | *BRCA1* | 17 | 32910537 | 32910538 | TC | - | frameshift deletion | NM_000059: exon 11, c.2049_2050del p.Ile684fs | 683 | 0.33 | class 5 | Pathogenic | rs80359319/Pathogenic |
|  |  | *BRCA2* | 13 | 41209079 | 41209079 | - | G | frameshift insertion | NM_007294: exon 20, c.5266dupC p.Gln1756fs | 811 | 0.41 | class 5 | Pathogenic | rs80357906/Pathogenic |
| A629 | IDC (32y) | *BRCA2* | 13 | - | - | exons 1-2 | - | gross deletion | NM_000059: exons 1-2, c.1-?_67+?del p.? | - | - | class 5 | Pathogenic | - |
| A630 | OSC (55y) | *BRCA1* | 17 | 41258504 | 41258504 | A | C | missense | NM_007294: exon 5, c.181T>G p.Cys61Gly | 314 | 0.50 | class 5 | Pathogenic | rs28897672/Pathogenic |
| A633 | DCIS (38y), IDC(45y) | *BRCA2* | 13 | 32944695 | 32944695 | G | A | splicing | NM_000059: exon 19, c.8487+1G>A | 116 | 0.58 | class 5 | Pathogenic | rs81002798/Pathogenic |
| A634 | IDC (40y) | *BRCA1* | 17 | 41209079 | 41209079 | - | G | frameshift insertion | NM_007294: exon 20, c.5266dupC p.Gln1756fs | 509 | 0.34 | class 5 | Pathogenic | rs80357906/Pathogenic |
| A643 | IDC (38y), OSC (70y) | *BRCA2* | 13 | 32912386 | 32912390 | TGAAA | - | frameshift deletion | NM_000059: exon 11, c.3894_3898delTGAAA p.Ile1298fs | 368 | 0.41 | class 4 | - | - |
| A651 | BC (35y) | *BRCA1* | 17 | 41219660 | 41219664 | ATTAG | - | frameshift deletion | NM_007294: exon 17, c.5035_5039del p.Leu1679fs | 337 | 0.39 | class 5 | Pathogenic | rs80357623/Pathogenic |
| A655 | OSC (51y), IDC (53y) | *BRCA1* | 17 | 41228505 | 41228505 | C | A | missense | NM_007294: exon 14, c.4484G>T p.Arg1495Met | 513 | 0.50 | class 5 | Pathogenic | rs80357389/Pathogenic |
| A677 | IDC (39y), IDC (45y) | *BRCA1* | 17 | 41244309 | 41244309 | A | T | nonsense | NM_007294: exon 11, c.3239T>A p.Leu1080Ter | 198 | 0.53 | class 5 | Pathogenic | rs80357145/Pathogenic |
| A678 | BMC (39y), IDC (47y) | *BRCA1* | 17 | - | - | exon 20 | - | gross deletion | NM_007294: exon 20, c.5194-?_5277+?del p.? | - | - | class 5 | Pathogenic | Pathogenic |
| A691 | IDC (53y) | *BRCA2* | 13 | 32907062 | 32907062 | - | CAGT | frameshift insertion | NM_000059: exon 10, c.1448_1451dupCAGT p.Lys485fs | 528 | 0.30 | class 5 | - | rs886040366/Pathogenic |
| A696 | OSC (51y) | *BRCA1* | 17 | 41228505 | 41228505 | C | A | missense | NM_007294: exon 14, c.4484G>T p.Arg1495Met | 383 | 0.49 | class 5 | Pathogenic | rs80357389/Pathogenic |
| A708 | DCIS (70y) | *BRCA2* | 13 | 32910437 | 32910437 | C | T | nonsense | NM_000059: exon 11, c.1945C>T p.Gln649Ter | 558 | 0.53 | class 5 | - | rs398122735/Pathogenic |
| A726 | IDC (51y), ILC (61y) | *BRCA2* | 13 | 32930689 | 32930689 | A | - | frameshift deletion | NM_000059: exon 15, c.7561delA p.Ile2521fs | 572 | 0.40 | class 5 | Pathogenic | rs886040717/Pathogenic |
| A728 | BC (45y), IDC (56y) | *BRCA2* | 13 | 32954022 | 32954022 | - | A | frameshift insertion | NM_000059: exon 23, c.9097dupA p.Thr3033fs | 318 | 0.32 | class 5 | Pathogenic | rs397507419/Pathogenic |
| A741 | IDC (34y), BC (39y) | *BRCA1* | 17 | 41209079 | 41209079 | - | G | frameshift insertion | NM_007294: exon 20, c.5266dupC p.Gln1756fs | 177 | 0.36 | class 5 | Pathogenic | rs80357906/Pathogenic |
| A758 | ILC (34y) | *BRCA2* | 13 | 32914529 | 32914529 | A | T | nonsense | NM_000059: exon 11, c.6037A>T p.Lys2013Ter | 755 | 0.52 | class 5 | Pathogenic | rs80358840/Pathogenic |
| A762 | BMC (59y), IDC (70y) | *BRCA2* | 13 | - | - | exon 3 | - | gross deletion | NM_000059: exon 3, c.68-?_316+?del p.? | - | - | class 5 | Pathogenic | - |
| A763 | IDC (46y) | *BRCA2* | 13 | 32914174 | 32914174 | C | G | nonsense | NM_000059: exon 11, c.5682C>G p.Tyr1894Ter | 594 | 0.50 | class 5 | Pathogenic | rs41293497/Pathogenic |
| A768 | IDC (57y), OCCA (68y) | *BRCA1* | 17 | 41246698 | 41246698 | G | A | nonsense | NM_007294: exon 11, c.850C>T p.Gln284Ter | 621 | 0.53 | class 5 | Pathogenic | rs397509330/Pathogenic |
| A775 | IDC (41y) | *BRCA1* | 17 | 41222949 | 41222967 | TCTTCTGGGGTCAGGCCAG | - | frameshift deletion | NM_007294: exon 16, c.4964_4982del p.Ser1655fs | 265 | 0.45 | class 5 | Pathogenic | rs80359876/Pathogenic |
| T088 | ILC (47y) | *BRCA1* | 17 | 41197751 | 41197751 | G | A | nonsense | NM_007294: exon 24, c.5536C>T p.Gln1846Ter | 348 | 0.50 | class 5 | Pathogenic | rs80356873/Pathogenic |
| TR69 | IDLC (43y) | *BRCA2* | 13 | 32930609 | 32930609 | C | T | nonsense | NM_000059: exon 15, c.7480C>T p.Arg2494Ter | 721 | 0.45 | class 5 | Pathogenic | rs80358972/Pathogenic |
| TR86 | IDC (41y) | *BRCA1* | 17 | 41244057 | 41244067 | CTAGTATCTTC | - | frameshift deletion | NM_007294: exon 11, c.3481_3491del p.Glu1161fs | 340 | 0.46 | class 5 | Pathogenic | rs80357877/Pathogenic |

Gross deletions identified by MLPA miss information about start/end and VAF due to the different technique performed.

^a^ Tumor histotype: BC (breast cancer), BMC (breast medullary carcinoma), DCIS (ductal carcinoma in situ), IDC (invasive ductal carcinoma), IDLC (infiltrating ductal and lobular carcinoma), ILC (invasive lobular carcinoma), OAC (ovarian adenosquamous carcinoma), OCCA (ovarian clear cell adenocarcinoma), OPC (ovarian papillary cystadenocarcinoma), OSC (ovarian serous carcinoma).

^b^ Mutation nomenclature according to the *Human Genome Variation Society* (HGVS).

^c^ Variant Allele Frequency.

^d^ Mutation classification according to the *BRCA Share*, *Breast Cancer Information Core* (BIC) and *Leiden Open Variation Database* (LOVD) (VUS: variant of uncertain significance).

^e^ Mutation classification according to the *Single Nucleotide Polymorphism Database* (dbSNP) and *Clinical Variant* (ClinVar).
