# Supplementary material for: Multiple-gene panel analysis in a case series of 255 women with hereditary breast and ovarian cancer
Source: Oncotarget. 2017 Apr 3;8(29):47064–75. doi: 10.18632/oncotarget.16791 (PMC5564544; doi:10.18632/oncotarget.16791)
Supplement: Supplementary file 3 [file oncotarget-08-47064-s003.docx]

Supplementary Table S3: List of the pathogenic/likely-pathogenic mutations in extra-*BRCA* genes detected in 21 patients.

| **Sample ID** | **Cancer (age)^a^** | ***BRCA* status^b^** | **Gene** | **chr** | **start** | **end** | **ref** | **alt** | **Mutation type** | **HGVS^c^** | **depth** | **VAF^d^** | **IARC class** | **dbSNP/ClinVar^e^** | **esp6500^f^** | **1000g^f^** | **exac03^f^** |
| --- | --- | --- | --- | --- | --- | --- | --- | --- | --- | --- | --- | --- | --- | --- | --- | --- | --- |
| A243 | ILC (26y), IDC (45y) | - | *PALB2* | 16 | 23641307 | 23641308 | AT | - | frameshift deletion | NM_024675: exon 5, c.2167_2168del p.Met723fs | 596 | 0.44 | class 5 | rs587776416/Pathogenic | NA | NA | 0,00007 |
| A284 | IDC (39y) | + | *ERCC3* | 2 | 128030511 | 128030511 | T | - | frameshift deletion | NM_000122: exon 11, c.1757delA p.Gln586fs | 1031 | 0.44 | class 4 | - | 0,00048 | NA | 0,00017 |
| A414 | IDC (30y) | + | *FANCA* | 16 | 89862330 | 89862333 | GTGA | - | frameshift deletion | NM_000135: exon 11, c.987_990del p.Thr329fs | 387 | 0.46 | class 5 | rs772359099/Pathogenic | NA | NA | 0,00003 |
| A479 | IDC (37y) | - | *FANCL* | 2 | 58386928 | 58386928 | - | TAAT | frameshift insertion | NM_018062: exon 14, c.1096_1099dupATTA p.Thr367fs | 892 | 0.25 | class 4 | rs759217526/Pathogenic/VUS | 0,00248 | NA | 0,00283 |
|  |  |  | *PALB2* | 16 | 23646416 | 23646416 | A | T | nonsense | NM_024675: exon 4, c.1451T>A p.Leu484Ter | 667 | 0.50 | class 5 | rs786203714/Pathogenic | NA | NA | NA |
| A482 | IDC (37y), IDC (37y) | + | *BRIP1* | 17 | 59761412 | 59761415 | TCTT | - | frameshift deletion | NM_032043: exon 20, c.2992_2995del p.Lys998fs | 611 | 0.43 | class 4 | rs786203717/Pathogenic | NA | NA | NA |
| A485 | IDC (43y), OSC (51y) | - | *PPM1D* | 17 | 58740695 | 58740695 | T | - | frameshift deletion | NM_003620: exon 6, c.1600delT p.Phe534fs | 350 | 0.11 | class 4 | - | NA | NA | NA |
| A502 | IDC (59y), IDC (59y) | - | *PALB2* | 16 | 23641307 | 23641308 | AT | - | frameshift deletion | NM_024675: exon 5, c.2167_2168del p.Met723fs | 592 | 0.45 | class 5 | rs587776416/Pathogenic | NA | NA | 0,00007 |
| A519 | IDC (32y) | - | *FANCM* | 14 | 45667921 | 45667921 | C | T | nonsense | NM_020937: exon 22, c.5791C>T p.Arg1931Ter (p.Gly1906fs) | 531 | 0.47 | class 5 | rs144567652 | 0,00085 | 0,00060 | 0,00088 |
| A520 | ILC (48y) | - | *PALB2* | 16 | 23641218 | 23641218 | G | A | nonsense | NM_024675: exon 5, c.2257C>T p.Arg753Ter | 459 | 0,6 | class 5 | rs180177110/Pathogenic | NA | NA | 0,00003 |
| A526 | IDC (40y), IDC (40y) | - | *RECQL4* | 8 | 145740367 | 145740367 | A | - | frameshift deletion | NM_004260: exon 9, c.1573delT p.Cys525fs | 141 | 0.34 | class 4 | rs386833845/Likely pathogenic | NA | NA | 0,00023 |
| A530 | ILC (62y), ILC (62y) | - | *PALB2* | 16 | 23647332 | 23647332 | G | A | nonsense | NM_024675: exon 4, c.535C>T p.Gln179Ter | 787 | 0.49 | class 4 | - | NA | NA | NA |
| A531 | IDC (38y) | - | *MSH6* | 2 | 48027775 | 48027775 | A | T | nonsense | NM_000179: exon 4, c.2653A>T p.Lys885Ter | 849 | 0.45 | class 5 | rs587782593/Pathogenic | NA | NA | NA |
| A532 | DCIS (47y), OSC (54y) | - | *RAD51D* | 17 | 33430317 | 33430317 | G | A | nonsense | NM_002878: exon 8, c.694C>T p.Arg232Ter | 381 | 0.50 | class 5 | rs587780104/Pathogenic | NA | NA | 0,00001 |
| A544 | DCIS (55y), DCIS (55y) | - | *PALB2* | 16 | 23646724 | 23646727 | AAGA | - | frameshift deletion | NM_024675: exon 4, c.1140_1143del p.Ser380fs | 434 | 0.46 | class 4 | - | NA | NA | NA |
| A554 | IDC (59y), IDC (77y) | - | *ATM* | 11 | 108153550 | 108153554 | CTTAT | - | frameshift deletion | NM_000051: exon 25, c.3690_3694del p.Asn1230fs | 382 | 0.46 | class 4 | - | NA | NA | NA |
| A579 | IDC (38y) | - | *FANCI* | 15 | 89824444 | 89824456 | AAGTTGTTCTTCT | - | frameshift deletion | NM_001113378: exon 15, c.1425_1437del p.Gln475fs | 302 | 0.47 | class 4 | - | NA | NA | NA |
| A633 | DCIS (38y), IDC (45y) | + | *ATM* | 11 | 108143570 | 108143570 | C | A | nonsense | NM_000051: exon 22, c.3275C>A p.Ser1092Ter | 193 | 0.48 | class 4 | - | NA | NA | NA |
| A695 | IDC (74y) | - | *FANCL* | 2 | 58453913 | 58453913 | G | A | nonsense | NM_018062: exon 4, c.223C>T p.Gln75Ter | 204 | 0.42 | class 4 | - | NA | NA | NA |
| A767 | IMC (63y), IDC (72y) | - | *ATM* | 11 | 108213997 | 108213997 | - | CTGTC | frameshift insertion | NM_000051: exon 57, c.8318_8322dupCTGTC p.Thr2773fs | 816 | 0.27 | class 4 | - | NA | NA | NA |
| A790 | IDC (29y), IDC (36y) | - | *TSC2* | 16 | 2127626 | 2127627 | AG | - | frameshift deletion | NM_000548: exon 26, c.2865_2866del p.Gln955fs | 845 | 0.46 | class 4 | - | NA | NA | NA |
| A806 | DCIS (26y) | - | *SLX4* | 16 | 3639996 | 3640020 | GCAGCACAGCTTCGCTTCTTGGTGG | - | frameshift deletion | NM_032444: exon 12, c.3619_3643del p.Pro1207fs | 446 | 0.33 | class 4 | - | NA | NA | NA |
|  |  |  | *BRIP1* | 17 | 59763197 | 59763197 | T | - | frameshift deletion | NM_032043: exon 19, c.2905delA p.Asn969fs | 372 | 0.47 | class 4 | - | NA | NA | NA |

^a^ Tumor histotype: DCIS (ductal carcinoma in situ), IDC (invasive ductal carcinoma), ILC (invasive lobular carcinoma), IMC (invasive mucinous carcinoma), OSC (ovarian serous carcinoma).

^b^ *BRCA* mutational status: + (patient with pathogenic mutation in *BRCA1/2* genes), - (patient wild type for *BRCA1/2* genes)

^c^ Mutation nomenclature according to the *Human Genome Variation Society* (HGVS)

^d^ Variant Allele Frequency.

^e^ Mutation classification according to the *Single Nucleotide Polymorphism Database* (dbSNP) and *Clinical Variant* (ClinVar) (VUS: variant of uncertain significance).

^f^ Population frequencies present in Esp6500, 1000Genomes and Exac03 databases.
