# Supplementary material for: Multiple-gene panel analysis in a case series of 255 women with hereditary breast and ovarian cancer
Source: Oncotarget. 2017 Apr 3;8(29):47064–75. doi: 10.18632/oncotarget.16791 (PMC5564544; doi:10.18632/oncotarget.16791)
Supplement: Supplementary file 6 [file oncotarget-08-47064-s006.docx]

Supplementary Table S6: Clinical features and personal/family cancer history of BC patients with no pathogenic mutations, classified on the basis of PolyPhen HVAR and SIFT predictions.

| **BREAST CANCER (BC)** | **Probably benign by PolyPhen/SIFT** | **PolyPhen/SIFT non concordant** | **Probably damaging by PolyPhen/SIFT** | ***P*** |
| --- | --- | --- | --- | --- |
|  | ***N (%)*** | ***N (%)*** | ***N (%)*** |  |
| **N. of patients** | 77 | 23 | 62 |  |
| **Age at diagnosis, years** |  |  |  |  |
| Median Age [Min-Max] | 41 [26-79] | 48 [26-60] | 42 [25-79] | 0.421 |
| Missing | 0 | 0 | 0 |  |
| **Histotype** |  |  |  |  |
| In situ carcinoma | 9 (12.50) | 4 (18.18) | 3 (5.00) | 0.158 |
| Invasive ductal carcinoma | 43 (59.72) | 16 (72.73) | 46 (76.67) |  |
| Invasive lobular carcinoma | 10 (13.89) | 2 (9.09) | 5 (8.33) |  |
| Other invasive histotypes | 10 (13.89) | 0 (0.00) | 6 (10.00) |  |
| Missing | 5 | 1 | 2 |  |
| **Grading** |  |  |  |  |
| Well-differentiated | 8 (13.33) | 0 (0.00) | 9 (17.65) | 0.304 |
| Moderately differentiated | 29 (48.33) | 13 (65.00) | 23 (45.10) |  |
| Poorly differentiated | 23 (38.33) | 7 (35.00) | 19 (37.25) |  |
| Missing | 17 | 3 | 11 |  |
| **Stage** |  |  |  |  |
| 0 | 9 (14.75) | 4 (22.22) | 3 (6.00) | 0.213 |
| I | 36 (59.02) | 6 (33.33) | 30 (60.00) |  |
| II | 11 (18.03) | 5 (27.78) | 13 (26.00) |  |
| III-IV | 5 (8.20) | 3 (16.67) | 4 (8.00) |  |
| Missing | 16 | 5 | 12 |  |
| **Tumor invasiveness** |  |  |  |  |
| In situ | 9 (12.50) | 4 (18.18) | 3 (5.00) | 0.128 |
| Invasive | 63 (87.50) | 18 (81.82) | 57 (95.00) |  |
| Missing | 5 | 1 | 2 |  |
| **Ki-67** |  |  |  |  |
| High (≥14) | 31 (62.00) | 8 (57.14) | 33 (66.00) | 0.811 |
| Low (<14) | 19 (38.00) | 6 (42.86) | 17 (34.00) |  |
| Missing | 27 | 9 | 12 |  |
| **St Gallen subtype** |  |  |  |  |
| Luminal A | 11 (25.58) | 4 (30.77) | 11 (23.91) | 0.589 |
| Luminal B1 | 18 (41.86) | 7 (53.85) | 16 (34.78) |  |
| Luminal B2 | 5 (11.63) | 2 (15.38) | 10 (21.74) |  |
| Her2 positive | 5 (11.63) | 0 (0.00) | 2 (4.35) |  |
| Triple negative | 4 (9.30) | 0 (0.00) | 7 (15.22) |  |
| Missing | 34 | 10 | 16 |  |
| **Second BC** |  |  |  |  |
| No | 59 (76.62) | 20 (86.96) | 51 (82.26) | 0.504 |
| Yes | 18 (23.38) | 3 (13.04) | 11 (17.74) |  |
| Median Age [Min-Max] ^a^ | 62 [36-82] | 60 [57-75] | 55 [32-65] | 0.303 |
| **Second OC** |  |  |  |  |
| No | 76 (98.70) | 22 (95.65) | 60 (96.77) | 0.485 |
| Yes | 1 (1.30) | 1 (4.35) | 2 (3.23) |  |
| Median Age [Min-Max] ^a^ | 65 | 55 | 74.5 [72-77] | - |
| **Other tumors** |  |  |  |  |
| No | 69 (89.61) | 20 (86.96) | 54 (87.10) | 0.842 |
| Yes | 8 (10.39) | 3 (13.04) | 8 (12.90) |  |
| **BC/OC in I-degree relatives** |  |  |  |  |
| No | 30 (38.96) | 7 (30.43) | 17 (27.42) | 0.340 |
| Yes | 47 (61.04) | 16 (69.57) | 45 (72.58) |  |
| **BC/OC in I/II-degree relatives** |  |  |  |  |
| No | 14 (18.18) | 2 (8.70) | 10 (16.13) | 0.616 |
| Yes | 63 (81.82) | 21 (91.30) | 52 (83.87) |  |
| **Other cancers in I-degree relatives** |  |  |  |  |
| No | 51 (66.23) | 14 (60.87) | 35 (56.45) | 0.497 |
| Yes | 26 (33.77) | 9 (39.13) | 27 (43.55) |  |
| **Other cancers in I/II-degree relatives** |  |  |  |  |
| No | 25 (32.47) | 7 (30.43) | 20 (32.26) | 0.983 |
| Yes | 52 (67.53) | 16 (69.57) | 42 (67.74) |  |

^a^ Median age, in years, refers to age at second cancer diagnosis
