# Supplementary material for: Multiple-gene panel analysis in a case series of 255 women with hereditary breast and ovarian cancer
Source: Oncotarget. 2017 Apr 3;8(29):47064–75. doi: 10.18632/oncotarget.16791 (PMC5564544; doi:10.18632/oncotarget.16791)
Supplement: Supplementary file 7 [file oncotarget-08-47064-s004.docx]

Supplementary Table S4: Clinical features and personal/family cancer history of BC patients with BRCA1 and BRCA2 pathogenic mutations.

| BREAST CANCER (BC) | BRCA1-mutated | BRCA2-mutated | P |
| --- | --- | --- | --- |
| N (%) | N (%) |
| N. of patients | 23 | 24 |  |
| Age at diagnosis, years |  |  |  |
| Median Age [Min-Max] | 39 [26-57] | 38.5 [25-70] | 0.766 |
| Missing | 0 | 0 |  |
| Histotype |  |  |  |
| In situ carcinoma | 0 (0.00) | 3 (13.64) | 0.410 |
| Invasive ductal carcinoma | 18 (78.26) | 14 (63.64) |  |
| Invasive lobular carcinoma | 3 (13.04) | 3 (13.64) |  |
| Other invasive histotypes | 2 (8.70) | 2 (9.09) |  |
| Missing | 0 | 2 |  |
| Grading |  |  |  |
| Well-differentiated | 0 (0.00) | 0 (0.00) | 0.019 |
| Moderately differentiated | 3 (15.00) | 10 (52.63) |  |
| Poorly differentiated | 17 (85.00) | 9 (47.37) |  |
| Missing | 3 | 5 |  |
| Stage |  |  |  |
| 0 | 0 (0.00) | 3 (20.00) | 0.169 |
| I | 10 (55.56) | 5 (33.33) |  |
| II | 7 (38.89) | 5 (33.33) |  |
| III-IV | 1 (5.56) | 2 (13.33) |  |
| Missing | 5 | 9 |  |
| Tumor invasiveness |  |  |  |
| In situ | 0 (0.00) | 3 (13.04) | 0.233 |
| Invasive | 23 (100.00) | 20 (86.96) |  |
| Missing | 0 | 1 |  |
| Ki-67 |  |  |  |
| High (≥14) | 19 (90.48) | 17 (89.47) | 1.000 |
| Low (<14) | 2 (9.52) | 2 (10.53) |  |
| Missing | 2 | 5 |  |
| St Gallen subtype |  |  |  |
| Luminal A | 0 (0.00) | 1 (6.67) | 0.286 |
| Luminal B1 | 6 (31.58) | 7 (46.67) |  |
| Luminal B2 | 4 (21.05) | 3 (20.00) |  |
| Her2 positive | 0 (0.00) | 1 (6.67) |  |
| Triple negative | 9 (47.37) | 3 (20.00) |  |
| Missing | 4 | 9 |  |
| Second BC |  |  |  |
| No | 17 (73.91) | 18 (75.00) | 1.000 |
| Yes | 6 (26.09) | 6 (25.00) |  |
| Median Age [Min-Max] a | 46 [39-54] | 50 [37-70] | 0.830 |
| Second OC |  |  |  |
| No | 22 (95.65) | 23 (95.83) | 1.000 |
| Yes | 1 (4.35) | 1 (4.17) |  |
| Median Age [Min-Max] a | 68 | 70 | - |
| Other tumors |  |  |  |
| No | 22 (95.65) | 22 (91.67) | 1.000 |
| Yes | 1 (4.35) | 2 (8.33) |  |
| BC/OC in I-degree relatives |  |  |  |
| No | 9 (39.13) | 8 (33.33) | 0.766 |
| Yes | 14 (60.87) | 16 (66.67) |  |
| BC/OC in I/II-degree relatives |  |  |  |
| No | 7 (30.43) | 3 (12.50) | 0.168 |
| Yes | 16 (69.57) | 21 (87.50) |  |
| Other cancers in I-degree relatives |  |  |  |
| No | 19 (82.61) | 14 (58.33) | 0.111 |
| Yes | 4 (17.39) | 10 (41.67) |  |
| Other cancers in I/II-degree relatives |  |  |  |
| No | 12 (52.17) | 10 (41.67) | 0.564 |
| Yes | 11 (47.83) | 14 (58.33) |  |

a Median age, in years, refers to age at second cancer diagnosis
