# Supplementary material for: Multiple-gene panel analysis in a case series of 255 women with hereditary breast and ovarian cancer
Source: Oncotarget. 2017 Apr 3;8(29):47064–75. doi: 10.18632/oncotarget.16791 (PMC5564544; doi:10.18632/oncotarget.16791)
Supplement: Supplementary file 8 [file oncotarget-08-47064-s005.docx]

Supplementary Table S5: Clinical features and personal/family cancer history of OC patients with BRCA1 and BRCA2 pathogenic mutations.

| OVARIAN CANCER (OC) | BRCA1-mutated | BRCA2-mutated | P |
| --- | --- | --- | --- |
| N (%) | N (%) |
| N. of patients | 8 | 1 |  |
| Age at diagnosis, years |  |  |  |
| Median Age [Min-Max] | 50 [38-68] | 66 | - |
| Missing | 0 | 0 |  |
| Histotype |  |  |  |
| Serous carcinoma | 7 (87.50) | 0 (0.00) | - |
| Other malignant histotypes | 1 (12.50) | 1 (100.00) |  |
| Borderline tumors | 0 (0.00) | 0 (0.00) |  |
| Missing | 0 | 0 |  |
| Grading |  |  |  |
| Well-differentiated | 0 (0.00) | 0 (0.00) | - |
| Moderately differentiated | 1 (21.50) | 0 (0.00) |  |
| Poorly differentiated | 7 (87.50) | 1 (100.00) |  |
| Missing | 0 | 0 |  |
| Stage |  |  |  |
| 0 | 0 (0.00) | 0 (0.00) | - |
| I | 1 (14.29) | 0 (0.00) |  |
| II | 1 (14.29) | 0 (0.00) |  |
| III-IV | 5 (71.43) | 1 (100.00) |  |
| Missing | 1 | 0 |  |
| Tumor invasiveness |  |  |  |
| Borderline | 0 (0.00) | 0 (0.00) | - |
| Invasive | 8 (100.00) | 1 (100.00) |  |
| Missing | 0 | 0 |  |
| Second BC |  |  |  |
| No | 6 (75.00) | 1 (100.00) | - |
| Yes | 2 (25.00) | 0 (0.00) |  |
| Median Age [Min-Max] a | 58.5 [53-64] | - | - |
| Other tumors |  |  |  |
| No | 8 (100.00) | 1 (100.00) | - |
| Yes | 0 (0.00) | 0 (0.00) |  |
| BC/OC in I-degree relatives |  |  |  |
| No | 1 (12.50) | 0 (0.00) | - |
| Yes | 7 (87.50) | 1 (100.00) |  |
| BC/OC in I/II-degree relatives |  |  |  |
| No | 1 (12.50) | 0 (0.00) | - |
| Yes | 7 (87.50) | 1 (100.00) |  |
| Other cancers in I-degree relatives |  |  |  |
| No | 5 (62.50) | 1 (100.00) | - |
| Yes | 3 (37.50) | 0 (0.00) |  |
| Other cancers in I/II-degree relatives |  |  |  |
| No | 4 (50.00) | 1 (100.00) | - |
| Yes | 4 (50.00) | 0 (0.00) |  |

a Median age, in years, refers to age at second cancer diagnosis
