# Supplementary material for: Multiple-gene panel analysis in a case series of 255 women with hereditary breast and ovarian cancer
Source: Oncotarget. 2017 Apr 3;8(29):47064–75. doi: 10.18632/oncotarget.16791 (PMC5564544; doi:10.18632/oncotarget.16791)
Supplement: Supplementary file 9 [file oncotarget-08-47064-s007.docx]

Supplementary Table S7: Clinical features and personal/family cancer history of OC patients with no pathogenic mutations, classified on the basis of PolyPhen HVAR and SIFT predictions.

| OVARIAN CANCER (OC) | Probably benign by PolyPhen/SIFT | PolyPhen/SIFT non concordant | Probably damaging by PolyPhen/SIFT | P |
| --- | --- | --- | --- | --- |
| N (%) | N (%) | N (%) |
| N. of patients | 8 | 3 | 8 |  |
| Age at diagnosis, years |  |  |  |  |
| Median Age [Min-Max] | 46.5 [28-58] | 61 [34-66] | 48 [36-81] | 0.421 |
| Missing | 0 | 0 | 0 |  |
| Histotype |  |  |  |  |
| Serous carcinoma | 6 (75.00) | 2 (66.67) | 3 (37.50) | 0.371 |
| Other malignant histotypes | 2 (25.00) | 1 (33.33) | 2 (25.00) |  |
| Borderline tumors | 0 (0.00) | 0 (0.00) | 3 (37.50) |  |
| Missing | 0 | 0 | 0 |  |
| Grading |  |  |  |  |
| Well-differentiated | 1 (12.50) | 1 (33.33) | 0 (0.00) | 0.833 |
| Moderately differentiated | 1 (12.50) | 0 (0.00) | 0 (0.00) |  |
| Poorly differentiated | 6 (75.00) | 2 (66.67) | 5 (100.00) |  |
| Missing | 0 | 0 | 3 |  |
| Stage |  |  |  |  |
| 0 | 0 (0.00) | 0 (0.00) | 0 (0.00) | 0.088 |
| I | 4 (66.67) | 2 (66.67) | 0 (0.00) |  |
| II | 0 (0.00) | 0 (0.00) | 1 (20.00) |  |
| III-IV | 2 (33.33) | 1 (33.33) | 4 (80.00) |  |
| Missing | 2 | 0 | 3 |  |
| Tumor invasiveness |  |  |  |  |
| Borderline | 0 (0.00) | 0 (0.00) | 3 (37.50) | 0.166 |
| Invasive | 8 (100.00) | 3 (100.00) | 5 (62.50) |  |
| Missing | 0 | 0 | 0 |  |
| Second BC |  |  |  |  |
| No | 7 (87.50) | 3 (100.00) | 4 (50.00) | 0.181 |
| Yes | 1 (12.50) | 0 (0.00) | 4 (50.00) |  |
| Median Age [Min-Max] a | 62 | - | 54.5 [45-81] | - |
| Other tumors |  |  |  |  |
| No | 8 (100.00) | 3 (100.00) | 8 (100.00) | - |
| Yes | 0 (0.00) | 0 (0.00) | 0 (0.00) |  |
| BC/OC in I-degree relatives |  |  |  |  |
| No | 5 (62.50) | 1 (33.33) | 5 (62.50) | 0.689 |
| Yes | 3 (37.50) | 2 (66.67) | 3 (37.50) |  |
| BC/OC in I/II-degree relatives |  |  |  |  |
| No | 2 (25.00) | 1 (33.33) | 5 (62.50) | 0.344 |
| Yes | 6 (75.00) | 2 (66.67) | 3 (37.50) |  |
| Other cancers in I-degree relatives |  |  |  |  |
| No | 4 (50.00) | 2 (66.67) | 4 (50.00) | 1.000 |
| Yes | 4 (50.00) | 1 (33.33) | 4 (50.00) |  |
| Other cancers in I/II-degree relatives |  |  |  |  |
| No | 2 (25.00) | 2 (66.67) | 3 (37.50) | 0.580 |
| Yes | 6 (75.00) | 1 (33.33) | 5 (62.50) |  |

a Median age, in years, refers to age at second cancer diagnosis.
